# Exploring Risk Factors Affecting the Mental Health of Refugee Women Living with HIV

**DOI:** 10.3390/ijerph15102326

**Published:** 2018-10-22

**Authors:** Agata Vitale, Judy Ryde

**Affiliations:** 1 College of Liberal Arts (CoLA), Bath Spa University, Newton Park, Bath BA29BN, UK; 2 Trauma Foundation South West, Barrow Castle, Rush Hill, Bath BA22QR, UK; judy.ryde@barrowcastle.co.uk

**Keywords:** refugees women, HIV, mental health, stigma, discrimination

## Abstract

Little is known about how the intersection of being a forced migrant and living with HIV can contribute to the development or exacerbation of pre-existing mental conditions. This study is set in this context and it aims to explore specific risk factors affecting the mental health of refugee women living with HIV. A total of eight refugee women living with HIV took part in the study; they were individually interviewed, and their transcripts were thematically analyzed. The overall findings indicated that participants’ mental health was impaired by multiple stressors associated with their conditions, such as racial discrimination, HIV-related stigma, including from health professionals, loneliness, and resettlement adversities. These all represent threats to public health, as they discourage individuals from engaging with adequate health/mental health services. Despite their situation, participants had not received psychological interventions and their healthcare was reduced to managing the physical symptoms of HIV. Participants indicated their need to take part in group interventions that could promote their mental health and social recovery. These findings are relevant to raising awareness about the specific risk factors affecting refugee women living with HIV and to provide evidence for public health interventions based on this specific population’s need.

## 1. Introduction

Implementing effective strategies to promote the mental health of refugee women living with HIV represents a complex public health issue as it needs to address simultaneously the challenges that this specific population experience as forced migrants, as well as dealing with the physical/psychological sequalae of having contracted the virus. It is therefore imperative to identify specific risk factors to which refugee women living with HIV are exposed, and to provide evidence for the development and the implementation of effective mental health interventions that enable them to improve their living conditions.

### 1.1. Background

The Human Immunodeficiency Virus (HIV) is transmitted when infected body fluids are in contact either with mucous membranes or damaged tissues or are directly injected into the bloodstream from a needle [1]. Once in the system, HIV attacks and becomes part of the CD4 cells (a type of white blood cell in the immune system); when these cells multiply to fight infections, they make more copies of HIV, to the point that the body cannot react to opportunistic infections, cancers and diseases. This in turn can lead to AIDS (Acquired Immunodeficiency Syndrome), which represents the last stage of HIV infection [2].

Up until the mid-1990s, individuals with HIV could progress to AIDS within a few years, however, with the introduction of antiretroviral therapy (ART), mortality and the morbidity rates of individuals living with HIV have significantly improved [3] and those who start ART in a timely way and who comply with it adequately can have a long life expectancy [4]. However, according to recent statistics, out of the approximately 36.9 million individuals who are affected currently by HIV globally, only about 20.9 million are receiving adequate treatment [1]. HIV, therefore, represents one of the most destructive infectious disease epidemics in recorded history [5] and recent statistics indicated that, in 2016, it killed one million individuals [1]. This shows that, despite the enormous investment in the HIV response over the past 20 years which is paying off, the epidemics continues to pose serious public health threats in all regions and predominantly to the most vulnerable groups, including those who are affected by forced migration, gender based-violence, social inequalities, stigmatization, and discrimination [6,7]. Women and young girls (who overall represent more than half of the global population affected by HIV) are among the most vulnerable to contracting the virus in many communities, especially in the high-burden epidemics of sub-Saharan Africa [7]. HIV, therefore, remains a cause of death, particularly in disadvantaged populations, and as the World Health Organization (WHO) Draft global health sector strategies HIV, 2016–2021 [8] indicates, providing immediate and effective treatment will remain an important public health priority globally.

### 1.2. HIV, Gender, and Mental Health

HIV does not only attack and debilitate the immune system but, in turn, has detrimental consequences for individuals’ health and mental health [9,10,11,12,13,14]. Specifically, mood disorders, anxiety disorders, and post-traumatic stress disorder (PTSD) have been identified as the most prevalent mental health disorders among individuals living with HIV [15]. Depression in particular, seems to be twice as common in people living with HIV (especially when symptomatic) than in uninfected individuals [16,17].

Furthermore, self-harm and suicide attempts and/or completion rates are also high in individuals affected by HIV [18,19,20]. The high comorbidity of mental disorders in individuals living with HIV can be explained by many psychological and environmental risk factors, particularly in those from disadvantaged backgrounds, including unemployment, poor housing, food insecurity, stigma, and fear of disclosing their condition and of interacting with the health care system [21].

Furthermore, gender inequalities play a significant role not only in HIV transmission, but also in the course of HIV and its comorbidity rates with mental disorders [22].

In this regard, a PubMed search conducted by Orza et al. cited nearly 800 peer-reviewed articles about the high comorbidity of mental health disorders in women who are HIV positive [23]. Women living with HIV from disadvantaged backgrounds seem particularly at risk of having co-occurring mental disorders. This might be related to multiple gender-related risk factors, such as gender dynamics that result in power imbalances, marginalization from access to goods and opportunities which power ensures [22], unprotected sex, trading sex for money or other goods, having sex with high-risk partners, substance misuse gender-based violence [23,24,25], sexual and reproductive health complications, and human rights [26], their poor access and adherence to healthcare and medications [27], and stressors during pregnancy [28]. Above all, women of color living with HIV show particularly high rates of mental disorders, and this might be due to the cumulative effect of stigma, racial discrimination, poverty, and immigration status, if they are living abroad [29]. As expected, the comorbidity of HIV and mental disorders often leads to poor compliance and adherence to antiretroviral therapy [30] and/or to the lack of adequate engagement with relevant health/mental health services [31,32,33,34,35]. This, in turn, further reduces the quality of life of women affected by HIV, including poorer psychological adjustment to a chronic, progressive and life-threatening illness, worse HIV treatment adherence and outcomes [36,37], and an increased risk of HIV transmission [31,38].

### 1.3. HIV, Gender and Refugees

Due to the adverse pre-migratory conditions and often the adverse journey, refugees represent a high-risk group for contracting HIV [39]. This might be due to multiple risk factors, including the HIV prevalence in refugees’ country of origin, the lack of health services available to them [40,41], and the abuses and violence they might be exposed to during their journey and in refugee camps. HIV-related symptoms can be further worsened in refugee populations by the fact that some of the host countries might be overburdened by the impact of AIDS and, therefore, they can be unable or unwilling to provide adequate treatment for them [42]. Refugee women and girls might be at greater risk of contracting HIV (both in their country of origin and, in some cases in the host country) which might be due to gender inequalities [39]. There is, in fact, plenty of evidence indicating that refugee women are disproportionately impacted by economic, legal, cultural, and social disadvantages, food insecurity, unequal distribution of goods and by the destruction of the community and family structures that usually protect them [43,44]. Furthermore, refugee women are often the main target of all forms of violence in their country of origin, during the journey and in refugee camps [45]. Since the normal social safety nets are absent during conflicts, refugee women might be also forced to exchange sexual services for money, food, or protection.

Furthermore, rape is often used as a weapon of war and refugee women might be subject to sexual violence and exploitation in refugee settings [39,40]. Refugee women living with HIV might face several challenges during their resettlement process as they deal with their multiple health/mental health stressors associated with having contracted the virus, as well as trying to understand and navigate a new health system [46,47].

### 1.4. Refugees, Gender, and Mental Health

As for other refugee populations, refugee women’s mental health might be impaired by multiple pre and post-migratory psychological and environmental factors, including, in some cases, by being forced to leave their country of origin because of war, genocide, discrimination and the violation of human rights [48,49], they might be exposed to inhumane conditions during their journey [50,51], and/or they might be facing several cultural, economic and language barriers during their resettlement process [50,51,52,53,54,55]. Furthermore, among refugees, women are frequently identified in humanitarian reports as being particularly exposed to physical and mental health difficulties; refugee women might be at greater risk of violence in their country of origin and during their journey [56]. In addition, during their resettlement, refugee women might face several challenges associated with their gender, including carrying the burden of raising their children in a new country, adjusting to changes in family dynamics and to the new expectations about their roles [56,57]. In some cases, refugee women’s mental health might be impaired by the fact that they have lost their previous family and community support (which is essential in collectivist cultures) and they might be left on their own to adjust to their new gender-related roles [56].

However, despite the multiple risk factors, refugee women have often proved to be resilient and to adapt well to post-migratory stressors [58], especially if they perceive adequate community support in the host country [59].

### 1.5. Rationale for the Study

Despite the existing evidence on the link that mental health has with HIV, gender, and forced migration, little is known about how their intersection (i.e., being affected simultaneously by the sequelae of having contracted the virus as well as by the challenges associated with being refugees) might affect refugee women living with HIV. The current study therefore is set in this context and it aims is to provide some support to the scarce literature in this field. The objectives of this study are to explore the risk factors that refugee women living with HIV are exposed to, the type of support available to them and their view of taking part in a narrative, group-based intervention. This latter objective will provide the basis to the longer study that the authors will conduct at a subsequent stage with the same population by exploring the effectiveness of a group-based narrative intervention to support their mental health and social recovery.

For the purpose of this study *mental health* does not simply refer to the lack of psychological symptoms attached to a diagnostic category [60]; but, in line with the World Health Organization (WHO), it is considered *a state of wellbeing in which an individual realizes his or her own abilities, can cope with the normal stresses in life, can work productively and fruitfully* ([61], p. 1). In addition, within this study, *mental health promotion*, refers to *the process of enhancing the capacity of individuals and communities to take control over their lives and improve their mental health* [62].

Furthermore, in line with 1951 Geneva Convention [63], in this study, the term refugee refers to a person who has a ‘well-founded fear of being persecuted in his or her country of origin for reasons of race, religion, nationality, membership of a particular social group, or political opinion’. 

## 2. Materials and Method

Due to the exploratory nature of the research, a qualitative study design was employed [64,65]. This design is considered well suited for health and wellbeing investigations, as it offers a rich insight into the direct experience of individuals who are struggling with specific health/mental health conditions [66]. Furthermore, qualitative methods are considered highly appropriate when conducting research with refugees, as they allow the researcher to listen directly to their voices [67]. This enables researchers to build ‘a refugee centred perspective’ where the diverse narratives told by participants are organized to tell their collective story directly rather than this being filtered by stakeholders [68]. Specifically, for this study, semi-structured interviews were used; those are considered the most suitable method of data collection when investigating sensitive topics with refugee populations [69].

### 2.1. Materials

This consisted, in the following order, of: an information sheet, a consent form, a demographic form (to gather generic demographic details), and the interview script; this latter contains questions aiming at fostering discussion around the research topics, including of living with HIV, being a refugee and the type of health/ mental support available to them.

A digital recorder was used to record the interviews.

### 2.2. Ethical Considerations

Ethical approval was granted by the Ethics Committee from the first author’s institution. The interview process was in line with the British Psychological Society’s (BPS) guidelines for professional practice [70] and the Ethical Guidelines for Good Research Practice with Refugee Studies [71]. The ethical principles of *respect for persons*, *beneficence*, and of *informed consent* were observed [72,73].

In line with these ethical principles and prior to the interview, the overall process was explained to participants in detail, including the research aims and objectives, the confidentiality of the study, the freedom not to answer any questions that they did not feel comfortable with, and of withdrawing at any time without any consequences. 

Furthermore, it was essential to take extra ethical considerations for this specific study. Some refugees fear interviews, as they may recall traumatic memories of interrogations they could have had within their home countries, or, despite the interviews with immigration officers offering the possibility of gaining residence, it might be in fact distressing for some specific people to be in an interview situation [68,74].

To address this concern, the mental health implications for those who may have been traumatized through being exposed and/or witnessing horrifying events, were not approached in the study, even if this might mean that participants’ mental health was likely not to fully emerge from the data [75]. Furthermore, the contact details of specific organizations that could provide support to participants who might have found the experience of being interviewed distressing, were provided in the information sheet and they were restated verbally by the researcher prior to the interview.

### 2.3. Selection Criteria

These were: being female, aged over 18, being a forced migrant, having a level of English that could sustain a conversation and being able to give informed consent.

### 2.4. Accessing the Sample

All participants were recruited from those attending a non-profit organisation providing support to individuals *living* with HIV in the South West of England. Potential participants were identified and approached by a migrant key worker from this non-profit organisation to ascertain if they were willing to take part in the study. To facilitate this process, the first author provided the manager with a leaflet containing some generic information about the study, along with the researchers’ contact details. Individuals who showed an interest in the research and who fitted the selection criteria met the first author on an individual basis for more detailed information. Out of nine individuals who were asked to take part in the study, eight agreed and the one declined for family reasons. The size of this sample is in line with the literature in this field, which indicates that, in conducting research with refugees, having small samples is relatively normal, as refugees are a difficult to reach, mobile population [76,77]. A small sample is also considered suitable for explorative studies, as the thoroughness of the close investigation enhances the validity of in-depth inquiry [78].

### 2.5. Procedure

To facilitate participants’ comfort in taking part in the research [76], they were individually interviewed in a room free of distraction at the HIV organization from which they were recruited. However, it was made clear, through the ethic forms, that the study was not commissioned or linked in any manner to that organization. Prior to starting the interviews, participants were given the information sheet and were asked to sign the consent form. It was deemed important to help the interviewees talk freely about topics they raised, and additional questions were only asked to seek clarification, illustration, or further exploration [79]. The interviews took approximately 45 min each and were audio-recoded with the participants’ permission.

### 2.6. Data Analysis

Interview transcripts were analysed via thematic analysis. This method is not tied to a predetermined theoretical perspective and/or predefined ideas [80], but it rather situates the coding process in the realm of evidence [81] and it is considered well suited for health and wellbeing investigations [65]. The analysis was primarily conducted by the first author; however, to ensure its trustworthiness and consistency, the second author independently analysed a sample of transcripts by using a coding template to ensure inter code agreement [81,82]. There were not many differences between the analysis undertaken by the two authors, and they agreed on the final themes.

## 3. Results

### 3.1. Overview of the Sample

The sample for the study consisted of eight women aged between 30 and 55 years. Seven participants were from Africa and one from Jamaica. The length of time that participants lived in the UK varied between nine and 18 years. Six participants had already obtained their Leave to Remain (i.e., residency), while the remaining two had temporary leave status. In line with the UK National Health Service policy, all participants were entitled to and benefited from primary and secondary care (i.e., these are free to asylum seekers and refugees in the UK) [83].

Except for one participant (who stated that she had a partner but that they were not cohabitating), all remaining ones were single at the time of the interviews. Six participants had secondary level education, one primary and the remaining one had a tertiary level education. Six participants had adult children, and one had young children who lived with her. Except for one participant, who worked as a carer for older people, the remaining participants stated that they were currently not working, mostly for ‘health related reasons’. All participants stated that they were receiving treatment for their HIV, however, other than the practical and emotional support offered by the non-profit organization they linked to, none of them was in receipt of psychological treatment by psychologists, psychotherapists or counsellors within 12 months of the interviews.

### 3.2. Qualitative Analysis

Participants provided a rich description of how the intersection of being HIV positive and refugees affected their mental health, including facing HIV-related stigma, racial discrimination, isolation, and feeling disempowered by the relationship they had with some of the health professionals involved in their care. The themes are organised in a manner that could facilitate the understanding of participants’ struggles with being HIV positive, as well as with being refugees. The final analysis contains both broad and more focused themes, and this is in line with the specific analytic method employed [84].

**Theme 1: The intersection of the HIV related symptoms and the practical challenges of being refugees**

Participants indicated that the quality of their life was significantly impaired by dealing with the sequelae of having contracted the virus, as well as with the practical challenges they faced as refugees, including their struggle to meet basic needs and their poor living conditions.

In particular, the lack of suitable accommodation represented one of the main concerns for all participants. Due to their financial restrictions, as well as their HIV-related symptoms, they were in fact often confined indoors with very little to entertain themselves. For instance, when talking about her financial struggle, P.7 said: *I am so poor, but this month I don’t think I can pay the rent because I am not working*; whereas P.5 indicated: *You’re limited on what you can do because you’re a refugee sometimes*. Furthermore, when talking about her daily routine, P.6 (who lived in a small council flat, in a block that she described as quite dull and unpleasant), said: *If I am not working and I don’t have an appointment then I stay home. I don’t go anywhere … First thing I pray. And yes, that is it. Maybe I may do cleaning, cleaning and doing other things, it is just inside (the house).*

In addition, P.1 (who lived in a shared catholic accommodation, with 13 residents) indicated: *I’m worried that my heart can stop at any time … So, I’m always in the house … I am limited in the activities I can do. If there is no one in the house, I spend the day sleeping because there is nothing to do …* P.1 also indicated she had to live under strict catholic rules, including that she did not even have the freedom to choose the television programs she wanted to watch: *As a Community House we are not allowed to watch television … we watch a few movies not like action movies, they would be sort of like censored movies.*

Furthermore, when talking about her financial situation P.2 stated: *The money they give us is not enough to rely on a week. Because I have to use transport, we have to eat healthy.* Her conditions were also aggravated by the lack of predictability of her HIV related symptoms: *So, my days aren’t predictable. I can get up this morning and I just felt fine and I can wake up tomorrow morning and feel quite sick …* As a result, P.2 indicated that she was forced to spend most of her time, indoors, in a small hostel room that she shared with her two young children: *Sometimes I just find it very difficult just to … because now I’m in one room with my children, because I’m still bidding for a house, I just find it challenging, waking up in the morning, at night I don’t have my own time, I have to go to bed when they have to go to bed, I don’t sit there to just reflect on myself.*

Furthermore, when talking about how HIV-related symptoms affected her, P.8 said: *It’s painful for me, it’s not letting me do anything. If I stand too long it’s a problem. I need to be only on the bed.* She added that being confined in a home was quite challenging for her, as she shared an over-crowded accommodation with other female refugees: *I share the house with the other ladies, it is very difficult. You know people from different countries and it’s really difficult to manage … We don’t have privacy, no privacy in the house and very, very difficult you know it’s very difficult for me to manage.*

The intersection of dealing with the sequelae of the HIV, as well as facing practical challenges associated with their condition, seemed to affect participants’ mental health, as they mostly complained about anxiety, insomnia, fatigue and lack of motivation. In this regard, P.1 *I tend not to do a lot because of my condition, because I get tired too early;* Or, P.3, who indicated: *My sleep is not very good, so I wake up still tired and sometimes I want to stay in bed. I don’t feel like doing anything, lack of motivation;* and P.5, who stated: *I don’t feel like doing anything. I’m tired, fatigued, (I have) chest infections.*

**Theme 2: Care received by health professionals: ‘I’m the one carrying the body’**

Participants indicated that, despite their circumstances, the type of care they were receiving was mostly related to reducing the physical symptoms of HIV, rather than taking a holistic approach that could address both their health/mental health needs. This was well exemplified by P.3 who, when talking about her mental conditions said: *Medication, I get it from the GPs (General Practitioners). Psychological help, nothing really*. Participants had mixed experiences of receiving care from the health professionals. The only exception was represented by P.4, who indicated: *Yeah, health professionals, they are quite fine. They don’t treat me in any way different. They treat me quite fine, so I can express myself to them.* However, P.4 was elusive in explaining the reasons of her satisfaction. From her interview and her demographic form, it was difficult to understand the causes of her satisfaction, as it not did seem related to having a better situation than the rest of the sample.

The remaining participants indicated that their General Practitioner (i.e., family doctor), who they saw regularly for routine cheek-ups and for the referrals to secondary care, was quite supportive and empathetic towards them. For instance, P.1 said that she was able to build a relationship overtime with her General Practitioner and that she could talk freely about her concerns: *We have a very good communication with my GP, I feel more happier whenever I go and sit with her, just to talk to her, explain to her how I feel.* This was echoed by P.8, who indicated: *If I feel down, I have my GP who I can talk to;* Or, for instance, by P.2, who stated: *He (the General Practitioner) treats me well.*

However, participants felt that they were not treated with the same respect by other health professionals involved in their care. For instance, when they had to book a last-minute appointment at their family practice (which happened quite often because of their HIV-related symptoms) they felt disempowered by some of the General Practitioners on duty. This is well exemplified by P.1, who stated:

There are times when you book an appointment and you see just any other doctor, they don’t want to listen. There are times when I say, “You are not listening to me, you are telling me but I’m the one carrying the body that you’re working on and you need to listen to what I’m saying that I’m feeling. Yes, I know it’s part and parcel of the condition but at the same time, I’m not well, you have to listen that I’m saying I’m not well.” But sometimes they’ll say, “Oh there’s nothing else we can do.”

In addition, some participants felt that other health professionals (not from the HIV clinic) involved in their care, might fear contracting HIV from them. In this regard, P.1. said: *They (the doctors) think they might be at risk of getting HIV from me*. Or P.6 discussed how some health professionals reacted when she disclosed being HIV positive, for the fear of being contaminated: *When you mention you (to some doctors) are HIV sometimes their face drops. They are like careful in this and that way, so when I go, “Yes I am HIV but it is undetectable if you treat me”.*

In addition, P.6 talked about her struggle when disclosing to a dentist she was referred to that she was HIV positive. P.6 said that she indicated in the pre-assessment form that she was HIV positive and this generated fear of contracting the virus in the dentist. P.6 felt that the long wait after she handed back her form at the reception was due to the dentist not knowing how to handle her situation: *But as soon as I signed that on the form my time I waited was up to almost five hours. She (the dentist) could not touch me.* P.6 then explained*: It took time and I was the last one* (in the waiting room). *But I knew that it was not the first one that if they don’t understand about HIV.* P.6 indicated that she reported this episode to her key worker from the HIV organization, who then wrote a letter to the dental clinic to find out what happened: *I asked my support worker and she wrote to the dental clinic and they wrote back and said ‘Oh, we are very sorry, some people don’t know how to take this but we need to train them’.* This experience discouraged P.6 to return to that dental clinic: *So I never went back.*

**Theme 3: Facing multiple forms of intolerance**

Overall participants indicated that, they were exposed simultaneously to the stigma of living with HIV, as well as racism as refugees and discrimination as migrants. These sub-themes are described below.

### 3.3. Facing HIV Stigma: ‘It’s the Sickness of Shame’

All participants struggled with the stigma associated with living with HIV, first in their countries of origin (where they contracted the virus) and then in the UK. The stigma affected their self-esteem and led to isolation from their family and/or community networks. When talking about how the stigma of living with HIV affected women in their countries, participants indicated that this was mostly associated with them having low moral standards and with promiscuous sexual behaviour. Specifically, when talking about people’s attitude towards women living with HIV in her country, P.4 said: *If they (people in her country) discover that you’re HIV, they try to shun you and try to keep to their friends.* Or, P.5, explained how being HIV positive is considered a moral fault in her country:

Yeah, you’re perceived as like you’ve been not a good person for you to contract HIV. You’re made to feel like it’s your fault all the time and, unless you talk to someone and you hear their story, you don’t know how they got it and it’s a shame because we then paint it with just one brush, you’ve all been not looking after yourself or you’ve been sleeping around or yeah. So it’s really sad.

The feeling of shame because of the HIV was also echoed by P.3, who stated:

In Africa when somebody has got it, it’s a disgrace, it’s shame, you know, you have to be left to die and things like that. So when I was diagnosed with this sickness, I’m thinking okay, it’s sickness of shame and now I’m going to die, nobody will want me, nobody will want to talk to me.

Furthermore, participants indicated that the fear that disclosing their HIV condition in the UK could also lead to social isolation and with being negatively judged. This was well illustrated by P.2. when said:

There’s a stigma (in the UK), because I can’t just wake up in the morning and say to my fellow colleagues or people I walk around with that I’m HIV, no, I can’t. This is something they’ll try to isolate you, because it has ever happened to me, one of my friends just discovered that I was HIV positive, she stopped bringing her children to my house. So at the end of the day you don’t just wake up and say you’re HIV, there’s a lot of stigma around.

In addition, P.8 said that she was forced to leave the UK city where she lived for many years after her neighbours found out she was HIV positive *… Because I tried in XXX they start pointing with their finger, this lady she is HIV positive, nobody wants to be approachable. I say oh my god and I decided to change the city, so I can start.*

However, some participants indicated the stigma associated with being HIV positive was due, in part, to peoples’ lack of understanding and education about how the virus spreads, including its transmission. In this regard, P.3 stated: *But some people are still ignorant about these things … A lot of people don’t know. People that I know, there’s a lot of people I know, not like friends but we talk to, but they don’t know.*

Furthermore, P.6 indicated that she could not find a partner because men in general have a poor understanding of the HIV transmission and, as soon as she disclosed it, they ran away: *Because some people they don’t know how to go about this HIV because they think, ‘Oh HIV I am dying, that women gave me HIV.* In addition, P.2 stated that she was surprised about her experience of communicating to the police (who intervened in a domestic dispute) that she is HIV positive, as the police was not aware of the difference between HIV and AIDS: *I said yes then I did tell her I was HIV positive, and she looked at me, she threw down the pen and she said you’re HIV positive? Do you have AIDs? I said no, it is HIV positive.* However, P.6 said that the police were willing to find out more on HIV transmission: *So I had to start explaining to her what HIV positive was, how you live, then she said oh my god, I need to learn more about this.*

### 3.4. Feeling Unwelcomed: ‘You Are Not Supposed to Be Here’

All participants indicated that they were exposed to racism as refugees as well as discrimination as migrants living in the UK. Specifically, when talking about her situation as a refugee, P.3 indicated: *Up to now, I’m still struggling with that issue (being unwelcome because she is a refugee), but I hope it’s going to be okay.* Furthermore, P.5 said:

It’s hard and hard sometimes. Sometimes there are situations that always remind you that you’re a refugee … I mean once they know that you’re a refugee they sort of look look down upon you and they make you feel rubbish and nothing.

When talking about the discrimination she faces as a migrant, P.1 she said:

*They (people in general) are welcoming in a way, but there is still that thing, no one will be able to break it. People might say, “Oh, things are changing in the UK.” Things are not changing, people are suppressing what they think, but it’s still there, even racism. People might say, “Racism is not there anymore.” Racism is still there … you still have got, these negative things about immigrants and other people from different cultures.* She also described the discrimination she experienced from the residents in her community home: *Because even when I’m living in a Christian Community, you still have got, these negative things about immigrants and other people from different cultures.*

In addition, P.6 felt that the Brexit referendum increased discrimination towards migrants:

*It is after Brexit that is where I have seen, it is just becoming like wildfire because of Brexit, ‘You foreigners, you have to leave.’ I think that is what they thought when it was Brexit. As soon as it is Brexit everyone will have to go*. P.6 also described her experience of racial discrimination at work: *But people take it in the wrong way and they will make you uncomfortable. They will really make you uncomfortable. Sometimes at work yes you can ask someone, ’Look can I help you?’ And they say, ‘No I don’t want you, go back to your country.’*

**Theme 4: Building social networks: ‘I have nobody to go’**

The multiple risk factors significantly impaired their ability to integrate and to build social networks. This included the way that participants were exposed to the practical challenges of being refugees as well as their health conditions and being exposed to HIV related stigma, racism, and discrimination.

However, according to all participants, their sense of loneliness was only eased by the staff members of the HIV organization from where they were recruited. These professionals were described by participants as very understanding, supportive and helpful, including dealing with practical issues and in helping them in feeling valued. In this regard, for instance, P.2 stated: *… they are welcoming and they have a heart*; and P.3 said *… they make me feel like it’s nothing, you know, it’s nothing;* or, when talking about her key worker P.4 indicated: *For me even to get that flat it was my support worker she really worked hard*. For the rest, participants discussed the detrimental effects that their sense of solitude and isolation had on them. In this regard, P.2 stated: *If I don’t have an appointment then I stay home. I don’t go anywhere, I stay just indoors*. OR P.1 indicated: *So, I’m always in the house … Like some people organise to go out for drinks. I don’t go out for drinks because of my condition, I can’t walk very far, so I get left behind because I can’t do much.* In addition, P.8 said: *I have nobody to go to visit, I stay home doing nothing. That’s it..., I don’t have friends in XXX (name of the city where she lives)*. Or P.7 indicated: *Sometimes I can stay in the house for one week and I haven’t seen anybody. Everybody is busy … I don’t live with anybody and because of this situation (being HIV positive).* In addition, P.6 said that, because of her mental health conditions, she feared meeting people*: It is partly because of I have a mental health problem as well so I have so much anxiety meeting other people and because of what happened before.* As indicated, the lack of social relations did affect their mental health, and this was well illustrated by P.3, who stated: *The only thing I don’t want is to be by myself and because as soon as I’m alone I start thinking about a lot of things.*

**Theme 5: The need to take part in narrative based-interventions**

Participants indicated their need and willingness to take part in group-based interventions that could promote their mental health. This might be explained by several factors, including the fact that none of them was receiving any form of psychological intervention at the time of the interviews. Furthermore, participants indicated the need to take part in an intervention that could help them share stories with women who are in a similar situation. In this regard, P.2 said: *… to encourage us, we need encouragement, we need … encourage, to encourage us. you need to sit together, come together, sit, talk about how you feel, share it with somebody.* This feeling of having a safe space to share their experience and receiving support from women in their situation was echoed by the remaining participants. For instance, for P.7:

You see. I will not be taking because it’s not being alone, people also have the same problems and it’s like it will keep me going. I just want to keep myself freely to be a human being. I don’t want to sit isolated and just take care of this … To me, I wish I will meet people so I will be happy.

Or said P.6 *… and then maybe if I join a group I am seeing other woman and I will feel I am not the only one. It will motivate me, that is another way, that’s how I feel*. Furthermore, P.3 stated: *It (the intervention) sounded like fun. Something to do to get together to run away from thinking of problem … I try to be busy because once I’m alone I start thinking and I’m stressed.*

Additionally, for P.5, indicated that taking part in the intervention could help her, as well as other women in her situation, to be resilient and, as she indicated, not to be defined by the HIV:

I think for women living with HIV, one thing that they must always try and maintain is looking after themselves and you know, reminding themselves that they’re beautiful and making themselves look … Yes and worth it and you’ve still got a lot to give. HIV is not going to define who you are.

Furthermore, P.6 discussed how talking directly about her multiple traumas might be difficult for her and, therefore, she might benefit by taking part in an art-based intervention:

I used to go to a therapist because half of the time if I start saying how I feel and so on and so on it makes me umm, how shall I say it, emotional. Yes. So I would rather draw how I feel but some talk. So I draw how I feel at the end of the session they will ask either do I want to share? If I don’t want to share.

## 4. Discussion

Overall, the findings of this study indicated that participants were affected by the intersection of living with HIV and being refugees, including struggling with the sequalae of having contracted the virus, feeling disempowered by some of health professionals, not being able to meet their basic needs, being exposed to multiple forms of discrimination, and feeling isolated; all these risk factors seemed to impair their mental health, their ability to build social networks, and to integrate in the UK. Furthermore, participants indicated their willingness to take part in group-based psychological interventions that could promote their mental health, build resilience and strengthen their social connections.

The intersection of HIV and social and health inequalities, such housing and food insecurity, seemed to have affected participants mental health [21,23,24,25,26,27,28,29].

Housing in particular represented one of the main concerns for participants; this might represent a threat to their mental health, as they were deprived of living space and routine, and affected the individual’s sense of safety and overall sense of identity [85,86]. The meaning that refugees give to ‘home’ is extremely important at this difficult time when they are attempting to settle into the community, and/or when they feel detached from their previous networks [87,88]. In this regard, a study conducted by Vitale and Ryde [75] indicated that one of the main sources of stress for male refugees is not having a ‘home’ where they feel protected while they are starting to build their social networks, including the ability to invite people to their home, especially as they have very limited means of socializing outside their houses. This particularly applied to the participants of this study, as the lack of suitable accommodation has been proven to exacerbate the physical and psychological symptoms of their HIV [89], as the individuals find it difficult to manage their chronic conditions. They might struggle to follow medication schedules, to secure and prepare nutritious meals and to rest adequately [89,90]. This specific finding of this study, therefore, supports the need to further develop our understanding of the role that housing plays in women living with HIV from socio-economic disadvantaged background, including refugees [91].

Another finding that emerged from the analysis was that participants were exposed simultaneously to prejudice, racial discrimination and the stigma of living with HIV.

Participants in fact experienced prejudice as refugees, as they reported feelings *unwelcomed, looked down on*, and *like rubbish,* and as migrants. This represented a threat to their mental health and might have contributed to the exacerbation of their health/mental health conditions [92].

Participants were exposed to HIV-related stigma, which means that they are devalued and discriminated against based on actual or perceived HIV-positive serostatus [93]. They also specified that they contracted HIV in their countries of origin, where they said the virus is still considered a *sickness of shame* and *a cause of disgrace.* As expected, the internalized HIV stigma contributed to negative beliefs about themselves and to low self-esteem [94]. Participants indicated that women with HIV are discriminated against in their countries of origin because they are considered to have low morals and/or promiscuous sexual behaviour; this happens even though female refugees living with HIV often contract the virus as the result of gender-based violence [7,46,54]. As expected, the HIV-related stigma they experienced in their countries had devastating effects on their mental health, including for the fact that they belong to collectivist cultures where being singled out could also have consequences for their survival [43]. In addition, participants indicated that, because of the HIV-related stigma, they feared losing the already weak social networks that they were attempting to build in the UK and this also represented a risk factor to their mental health [94]. As expected, the intersection of racial discrimination, social inequalities, resettlement adversities and HIV stigma might lead participants to being socially isolated and disconnected [95], which, in turn, might have impaired their mental health [95,96,97].

Social ties and networks are in fact considered essential in promoting and maintaining good mental health [98]; this particularly applies to refugees, as being able to build networks increases their sense of belonging in the new country and therefore strengthens their new sense of identity [48,99,100]. In addition, social support, including emotional and instrumental assistance has been shown to promote better health and to buffer against the negative effects of stressors on health in individuals living with HIV [101], including increased coping skills and treatment success [102].

As participants suggested, to reduce isolation for individuals living with HIV, it is important to educate others, including health professionals, about the virus and its transmission. Health services in fact represent one of the main settings for HIV-related stigma and this might be due to a lack of awareness in health professionals of the importance of not perpetuating stigma and/or their fear that casual contact with patients might pass on the disease [103].

HIV-related stigma from health professionals therefore represents a significant public health concern and is one of the main barriers to individuals with HIV accessing adequate treatment [93].

Furthermore, when talking about barriers to accessing healthcare, participants indicated that, they felt disempowered by the relationship they had with some of their health professionals, and particularly from those who were not directly involved in their HIV care. This indicates the importance of health professionals listening to the refugees’ voice. Empowering individuals to make decisions about their own care is considered one of the key factors in supporting the recovery of individuals experiencing mental distress [104,105,106]; it is also in line with the WHO Global Health Strategy [8] which recommends enabling individuals with HIV to be important partners in their treatment.

However, the findings from the current study indicated that the participants felt empowered and did have a voice in the care provided by their General Practitioners; this supports the need to provide adequate support for General Practitioners in their role of managing patients with chronic diseases [107,108]. This is also in line with the current WHO: Global Health Sector Strategy on HIV [8], which stresses the need for individuals living with HIV to have a continuum of care across health services, starting with primary care.

Another finding from this study is that, despite the need for mental health interventions, participants were simply receiving treatment for the health conditions associated with HIV. This represents a public health concern, as there is plenty of evidence which indicated that poor mental health also leads to physical health conditions and faster disease progression [13,14,109,110,111], as it is becoming increasingly clear that physical health cannot be detached from psychological well-being [15]. It is important, therefore, that refugee women living with HIV have appropriate access to comprehensive HIV treatment, care, and support [7,8].

The crossing of multiple adversities that participants experienced, therefore, might have aggravated participants’ mental health, which might have been already impaired by the pre-migratory risk factors that generally affect refugees [49,50,51,52,53], as well as the trauma of having contracted HIV through gender-based violence [22].

It is, therefore, important to provide effective care for refugee women living with HIV, which has a holistic approach to their health/mental health needs and can foster their social recovery [112], and community integration [113]. In turn, social recovery promotes, cohesion, their personal and social identity [104,105], and promote resilience in refugee women [60]. This is in line with the findings of the current study, which indicated that participants were willing to take part in interventions that could support their ability to cope with their condition as well as their social recovery, and their ability to share their experience and build cohesiveness with other individuals in their situation. These factors are considered essential in supporting in the quality of life and the overall social recovery following a diagnosis of HIV [114,115].

## 5. Conclusions

The risk factors affecting refugee women living with HIV are complex as these are strengthened by the intersection of the health and psychological sequalae of having contracted the virus, as well as the poor living conditions which are often associated with being forced migrants. This is an extremely vulnerable population, as they might be exposed to social and gender inequalities, as well as racial discrimination as refugees and migrants, and HIV-related stigma, including in health care settings. The risk factors they experience represent a threat to public health, as it might discourage refugee women living with HIV from seeking adequate treatment. In line with the current WHO Global Health Strategy on HIV, the findings of the current study suggest that the emphasis of the treatment and care of these individuals should shift from the management of the physical symptoms of HIV, to comprehensive and multidisciplinary care that addresses the multiple risk factors associated with their conditions. In addition, refugee women living with HIV should have a voice in decisions about their care and they should be involved in interventions that promote their mental health, integration, and their overall social recovery.
